# A New Year

**Published:** 1853-01

**Authors:** 


*A New Year.*—A happy new year to our patrons. "May their shadow never be less!" May the present be fraught to them with more happiness than any year that has past and with less than any that is to come.

We congratulate them upon the pleasures of January. How great is the satisfaction of gathering in the rewards of labor, sweetened as is the reception of fees with the consciousness of deserving them! We congratulate them upon the other pleasure of paying debts. How pleasant it is to remove from our consciences all sense of pecuniary obligation, and see the gratification of the removal multiplied in the comfortable feelings of the recipient of the golden representatives of value. We do not like to use what is not paid for. Whatever it is, it is a tireless dun, ever obtruding itself at unwelcome times. It is dreadful to know that the baker has a lien on our stomach, and the tailor upon our back, and the publisher upon our brains! Dear friends, we have pledged our consciences to the printer for mental aliment for you. This you have received, and inwardly digested, and it has become part and parcel of yourselves. According to Paley, a man acquires a right to that with which his labor is mixed, and it grieves us to think that we have a lien or mortgage moral upon any of our fellow citizens. We trust, therefore, that such of our readers as are in arrears to the Journal will redeem themselves and be free intellectually; knowing that their thoughts are honestly their own, and enable us to redeem our honor pledged now to the printer, so that we too may feel our liberty restored.

Again, dear friends, we wish you a happy new year—an enjoyment in which our inexorable printer, though a very good natured man, will not permit us to participate until we have paid him to the uttermost farthing.

